# Exploration of the Mechanism of Lianhua Qingwen in Treating Influenza Virus Pneumonia and New Coronavirus Pneumonia with the Concept of “Different Diseases with the Same Treatment” Based on Network Pharmacology

**DOI:** 10.1155/2022/5536266

**Published:** 2022-02-08

**Authors:** Huihui Su, Guosong Wu, Lulu Zhan, Fei Xu, Huiqin Qian, Yanling Li, Ximei Zhu

**Affiliations:** ^1^College of Pharmacy, Sanquan College of Xinxiang Medical University, Xinxiang 453000, China; ^2^Department of Pharmacy, Baiyun Branch of Nanfang Hospital of Southern Medical University, Guangzhou 510599, China; ^3^Clinical Pharmacists, The Maternal and Child Health Care Hospital of HuaDu District (Huzhong Hospital), Guangzhou 510800, China

## Abstract

The 31 main components of Lianhua Qingwen (LHQW) were obtained through a literature and database search; the components included glycyrrhizic acid, emodin, chlorogenic acid, isophoroside A, forsythia, menthol, luteolin, quercetin, and rutin. Sixty-eight common targets for the treatment of novel coronavirus pneumonia (NCP) and influenza virus pneumonia (IVP) were also obtained. A “component-target-disease” network was constructed with Cytoscape 3.2.1 software, and 20 key targets, such as cyclooxygenase2 (COX2), interleukin-6 (IL-6), mitogen-activated protein kinase14 (Mapk14), and tumor necrosis factor (TNF), were screened from the network. The David database was used to perform a Kyoto Encyclopedia of Genes and Genomes (KEGG) signal pathway enrichment analysis and gene ontology (GO) biological process enrichment. Results showed that the key targets of LHQW in the treatment of NCP and IVP mainly involved biological processes, such as immune system process intervention, cell proliferation, apoptosis and invasion, toxic metabolism, cytokine activity, and regulation of the synthesis process. KEGG enrichment analysis revealed that 115 signalling pathways were related to the treatment of LHQW. Amongst them, IL-17, T cell receptor, Th17 cell differentiation, TNF, toll-like receptor, MAPK, apoptosis, and seven other signalling pathways were closely related to the occurrence and development of NCP and IVP. Molecular docking showed that each component had different degrees of binding with six targets, namely, 3C-like protease (3CL), angiotensin-converting enzyme 2 (ACE2), COX2, hemagglutinin (HA), IL-6, and neuraminidase (NA). Rutin, isoforsythiaside A, hesperidin and isochlorogenic acid B were the best components for docking with the six core targets. The first five components with the best docking results were isoforsythiaside, hesperidin, isochlorogenic acid B, forsythin E, and quercetin. In conclusion, LHQW has many components, targets, and pathways. The findings of this work can provide an important theoretical basis for determining the mechanism of LHQW in treating NCP and IVP.

## 1. Background

Lianhua Qingwen (LHQW) is composed of 13 traditional Chinese medicines (TCMs), namely, Forsythiae Fructus (Lianqiao, LQ), Lonicerae Japonicae Flos (Jinyinhua, JYH), Ephedare Herba (Mahuang, MH), Armeniacae Semen Amarum (Kuxingren, KXR), Gypsum Fibrosum (Shigao, SG), Isatdis Radix (Banlangen, BLG), Dryopteridis Crassirhizomatis Rhizoma (Mianmaguanzhong, MMGZ), Houttuyniae Herba (Yuxingcao, YXC), Pogostemonis Herba (Guanghuoxiang, GHX), Rhei Radix et Rhizoma (Dahuang, DH), Rhodiolae Crenulatae Radix et Rhizoma (Hongjingtian, HJT), l-Menthol (Bohenao, BHN), and Glycyrrhizae Radix et Rhizoma (Gancao, GC). According to the pathological theory of TCM, LHQW is good medicine for dredging collaterals, dispersing the lung, opening with pungent and bitter drugs, and clearing away heat and detoxification [1]. It exerts good effects on influenza virus pneumonia (IVP) and has antibacterial, antipyretic, anti-inflammatory, antitussive, expectorant, and immune-regulating functions [2]. LHQW has no difference with oseltamivir [3] (oseltamivir is a neuraminidase inhibitor used in the treatment of influenza A and B) in terms of anti-influenza capability, but it is superior to oseltamivir in relieving the symptoms of influenza, especially fever, cough, headache, muscle soreness, and fatigue. It can also block the vicious cycle of multiple pathological links and plays a role in overall regulation and multitarget treatment. In addition, LHQW, as an effective drug for novel coronavirus pneumonia (NCP) diagnosis and treatment (Trial Seventh Edition issued by China in 2019), exerts a significant clinical effect on the treatment of coronavirus disease 2019 (COVID-19). Multiple retrospective analyses have shown that LHQW can significantly improve the clinical symptoms of fever, cough, expectoration, shortness of breath, and other common symptoms experienced by patients diagnosed with COVID-19; it can also alleviate the disease's condition and shorten its course [4–6]. LHQW has a significant effect on the treatment of IVP and NCP, but no systematic study has been conducted on the molecular mechanism of LHQW treatment of the two diseases.

Given the complexity of the composition and mechanism of traditional Chinese medicine (TCM), systematically explaining the mechanism of TCM in the treatment of diseases is difficult. Network pharmacology is a new technology that integrates system biology, multidirectional pharmacology, and computer analysis. It can systematically link drugs with diseases and observe the intervention and influence of drugs on disease networks [7–9]. The systematicness of network pharmacology coincides with the integrity of TCM in treating diseases.

In recent years, network pharmacology has been widely used to systematically explain the mechanism of action of TCM and TCM compound prescription in the treatment of diseases [10–12], and extensive research has been conducted on the “different diseases with the same treatment” concept of TCM [13]. Examples include the mechanism of brain and heart treatment of Danhong injection [14]; the mechanism of network pharmacology of Jiaotai pills for diabetes, depression, and insomnia [15]; the mechanism of network pharmacology of Shengmai Yin in treating diabetes and heart failure [16]; and analysis of Chaihu Guizhi decoction based on the network pharmacology model for the treatment of gastric ulcer and epilepsy [17]. In this work, we aim to use a comprehensive network pharmacology-based approach to investigate the mechanism of how LHQW exerts therapeutic effects on viral pneumonia.

## 2. Materials and Methods

### 2.1. Retrieval of the Main Components of LHQW and Prediction of Its Target

The main components of LHQW can be identified and quantified through literature retrieval. Through verification and conversion of the PubChem database (https://pubchem.ncbi.nlm.nih.gov/), the target protein of the compound was obtained from the Traditional Chinese Medicine Systems Pharmacology (TCMSP) database (http://tcmspw.com/tcmsp.php), National Center for Biotechnology Information (NCBI) database (https://pubchem.ncbi.nlm.nih.gov/), and https://dx.doi.org/10.1093/nar/gkz382 (SWISS) database (http://www.swisstargetprediction.ch/). At the same time, the protein database UniProt (http://www.uniprot.org/uploadlists/) was used to convert the targets to a unified gene name.

### 2.2. Identification of Antiviral Pneumonia-Related Targets of LHQW

GeneCards database (https://www.genecards.org/) is a platform that provides all known human genes in genome, proteome, transcription, heredity, and function [18]. NCP- and IVP-related target information were collected by searching for the keywords “influenza virus pneumonia” and “novel coronavirus pneumonia.” The IVP-associated targets, NCP-related targets, and compound targets were mapped in Venny 2.1.0 software, and the common targets were screened out and regarded as a potential target for LHQW to fight viral pneumonia.

### 2.3. Construction of “Native Component-Target” Network and Screening of Key Targets

To determine the interaction between viral pneumonia potential therapeutic targets and targets of LHQW, this study introduced the selected targets into the STRING network platform (https://string-db.org/) to construct a protein-protein interaction (PPI, the process of combining two or more proteins to determine their biochemical functions) network map. The database parameter was set to “Homo sapiens,” and the protein type was set to the rating criterion (confidence level >0.9). Then, the PPI network file was downloaded. The active components, their corresponding LHQW targets, and PPI were input into Cytoscape 3.2.1 to construct the “native component-target” network of LHQW. The topology parameters of the network were analysed using the network analyser function of Cytoscape and derived. The related topological data, such as degree (DG) and betweenness centrality (BC), were examined. DG and BC are important topological parameters for evaluating the specific gravity of a node in the network. DG can reflect the number of links between one node and other nodes in the network, and BC reflects the ratio of the number of paths passing through the node to the total number of shortest paths in all the shortest paths in the network. The larger the topology parameters of the node are, the more critical the target in the network is [19, 20].

### 2.4. KEGG Signal Pathway and GO Biological Process Enrichment Analyses

The core targets of LHQW in the treatment of viral pneumonia were subjected to gene ontology (GO) biological process and Kyoto Encyclopedia of Genes and Genomes (KEGG) signal pathway enrichment analyses by using the David database (https://david.ncifcrf.gov/). GO enrichment can be used to understand the main action process of the target. The KEGG pathway can be adopted to understand the role of this target in metabolism, signal transduction and other pathways by observing the distribution of targets in the pathway. The main signalling pathways and biological processes involved in the efficacy of LHQW were obtained by screening the target genes with *P* < 0.05. The *P* value is the statistical significance of target enrichment/annotation, and *P* < 0.05 indicates that the pathway has analytical significance. The smaller the *P* value is, the more significant the enrichment result is.

### 2.5. Molecular Docking and Data Processing

**Figure 1:**
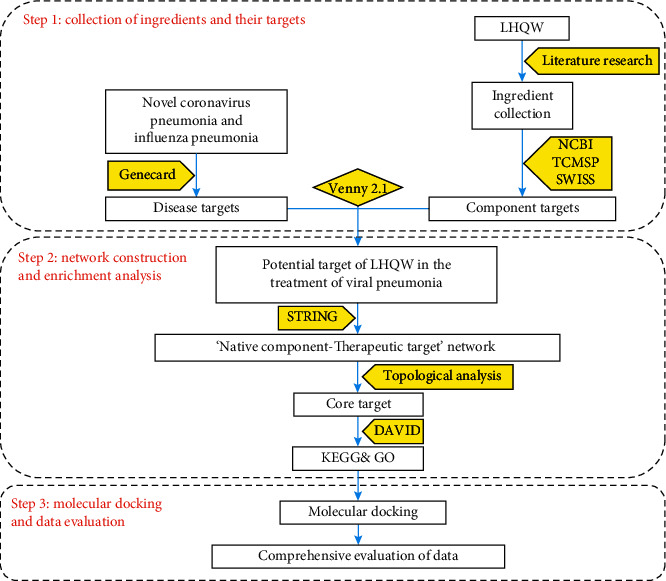
Flowchart of the research.

The protein structure file was downloaded from the Protein Data Bank (PDB) website (https://www.rcsb.org/), and the target protein and component were pretreated using AutoDock 1.5.6 and Discovery Studio 2.5 software. Then, the target and component were docked using AutoDock Vina. Generally, negative values of binding energy are associated with the stable conformation of ligand binding to the receptor. If the binding energy is less than −5 kcal/mol, then the ligand and receptor can bind spontaneously; binding energy ≤−7 kcal/mol denotes good binding [21, 22]. The flowchart of the experimental procedures of our study is shown in Figure 1.

## 3. Results

### 3.1. Main Components and Corresponding Targets of LHQW

After searching previous studies [23–26], we obtained 31 active components that could be detected qualitatively and quantitatively in LHQW. The composition analysis of LHQW shown above indicated that Lonicerae Japonicae Flos had 10 chemical constituents, Forsythiae Fructus had 9, Pogostemon Cablin (Blanco) Benth had 6, and Isatdis Radix had 4. Ephedra Herba, Houttuyniae Herba, Radix Rhei et Rhizome, and Licorice each had 3 chemical constituents. Menthae Herba had 2 chemicals, and *Rhodiola rosea* L. and Amygdalus Communis Vas each had 1 chemical constituent (Table 1).

In this study, 104, 414, and 341 predicted targets were obtained from TCMSP, NCBI, and SWISS databases, respectively. After UniProt correction and elimination of duplicate items, a total of 712 targets related to LHQW were obtained (Table 2).

### 3.2. Identification of Potential Targets of LHQW in Fighting Viral Pneumonia

**Figure 2:**
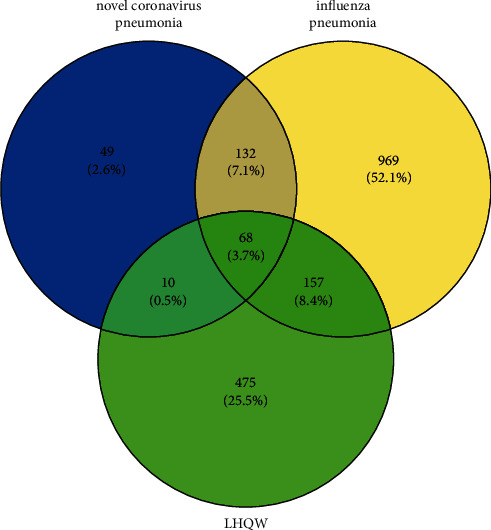
Venny figure.

The NCP- and IVP-related targets were derived from the GeneCards database by using “novel coronavirus pneumonia” and “influenza virus pneumonia” as the keywords, respectively. The NCP- and IVP-related targets from the GeneCards database numbered 259 and 1326, respectively. These targets were mapped in Venny 2.1.0 to map the 68 common targets (Figure 2). These targets are common targets related to the development of NCP and IVP, and they are the reason why LHQW can be used to treat NCP and IVP. The other NCP-related targets are 3C-like protease (3CL) and angiotensin-converting enzyme 2 (ACE2). The most representative IVP targets are neuraminidase (NA) and hemagglutinin (HA). They embody the characteristics of seeking common ground whilst reserving differences in the process of “plague,” as indicated in TCM.

Potential target of LHQW intervention for viral pneumonia (yellow circle: the larger the topology parameter is, the larger the target circle is). Main ingredients of LHQW (red triangle) and medicinal composition of LHQW (green diamond).

### 3.3. Construction and Analysis of the “Native Component-Target” Network

**Figure 3:**
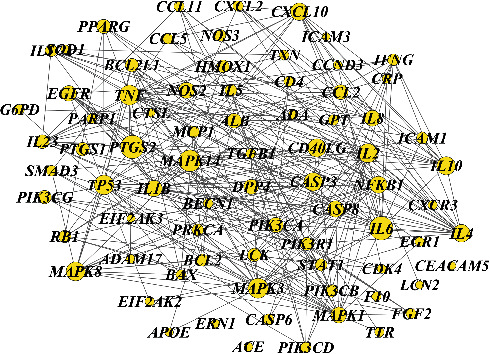
PPI network of LHQW against viral pneumonia. Novel coronavirus pneumonia target (purple), influenza pneumonia target (yellow), and LHQW component target (green).

**Figure 4:**
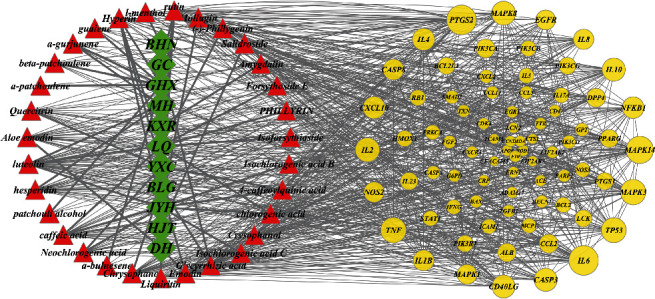
“Herb-component-target” network of LHQW in the treatment of viral pneumonia.

To further explore the mechanism of LHQW, 68 target proteins of LHQW against viral pneumonia were input into the STRING database, and the PPI network file was obtained (Figure 3). The medicinal materials, components, and PPI network were imported into Cytoscape software to construct the “herb-component-target” network of LHQW against viral pneumonia (Figure 4). The network has 109 nodes (including 31 compound nodes, 68 target protein nodes, and 11 kinds of medicinal materials) and 222 edges. Each candidate compound acts on an average of 17.4 targets, and the average number of drugs linked to each target protein is 6.6, indicating that LHQW has the characteristics of multicomponent and multitarget in the treatment of diseases. In combination with the analysis of network topology, the DG and BC of a network node are common indicators to describe the importance of the node. The median of DG in the targets is 20, and the median of BC in the targets is 0.003. A total of 20 nodes are more than twice the value and twice the center of this value (Table 3), suggesting that these targets are also the key factors to LHQW's treatment of viral pneumonia.

### 3.4. GO Biological Process Analysis

**Figure 5:**
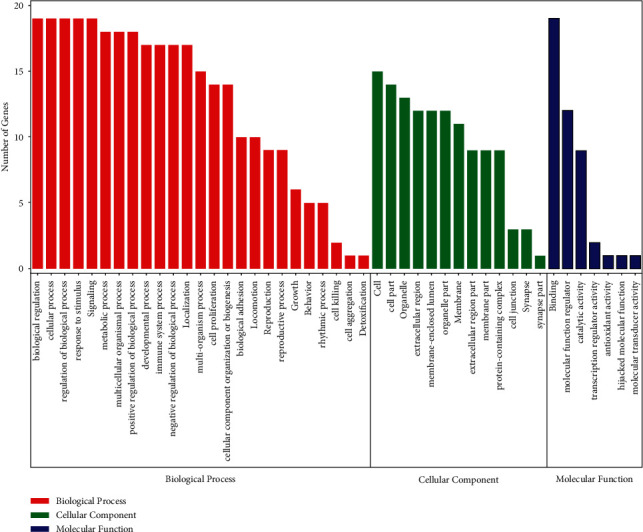
GO biofunctional notes. Biological process (red), cellular component (green), and molecular function (blue).

Twenty key targets were annotated with GO terms, and 91 terms with *P* < 0.05 were screened out. In general, the GO terms were classified into three parts: biological process (BP), molecular function (MF), and cellular component (CC). The visualisation results of BP, CC, and MF enrichment analyses were obtained using OmicShare, as shown in Figure 5. These targets are mainly distributed in the extracellular matrix, intercellular synapse, membrane sealing cavity, organelles, membrane system, and other cellular components. They play roles in molecular functions, such as molecular function regulation, binding, antioxidant activity, catalytic activity, and transcriptional regulation activity and participate in immune system intervention, cell proliferation, cell apoptosis, cell invasion, toxicant metabolism cytokine activity, and synthesis process regulation. These results suggest that LHQW functions against viral pneumonia by intervening in the above-mentioned biological processes.

### 3.5. KEGG Signal Pathway Enrichment Analysis

**Figure 6:**
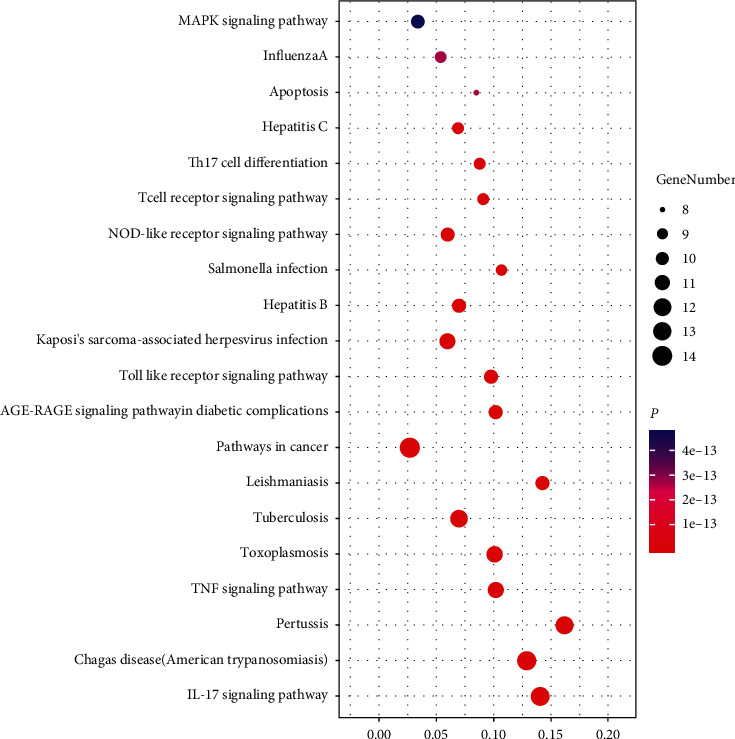
KEGG pathway map. The larger the bubble is, the more the targets enriched in the pathway are; the darker the bubble is, the more significant the enrichment pathway is.

The enrichment analysis of KEGG signalling pathways was carried out on 20 key targets, and 115 signal pathways with *P* < 0.05 were selected. The visualisation results of the top 20 KEGG pathways with the most significant *P* value were obtained using OmicShare, as shown in Figure 6. These pathways are mainly involved in immunity (T cell receptor/IL-17/Th17 cell differentiation signalling pathway), inflammation (TNF/toll-like receptor (TLR) signalling pathway), signal transduction (MAPK signalling pathway, etc.), cell apoptosis (apoptosis signalling pathway), and other pathways involved in influenza A, B, and C viral hepatitis. These results suggest that LHQW may play an integral role in the treatment of viral pneumonia by intervening in many pathways related to immunity, inflammation, viruses, and so on.

### 3.6. Molecular Docking

**Figure 7:**
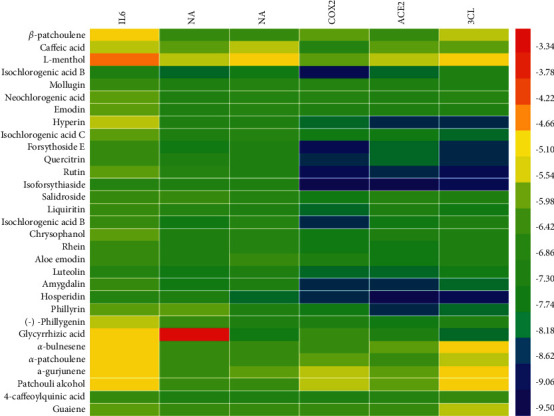
Heatmap of binding energy. The scale on the right represents binding energy, and the colour from red to blue represents the binding energy value arranged from large to small.

The occurrence and development of a new coronavirus and influenza virus exhibit similarities and differences after infection. The former mainly involves two targets (3CL and ACE2) [27–30], whereas the latter is mainly related to NA and HA [31–33]. Although the initial routes of infection are different, both cause a cytokine storm and further worsen the patient's condition [34, 35]. According to the results of this study, COX2 and IL-6 are involved in the infection. Therefore, this study further explored the material basis of LHQW in the treatment of viral pneumonia. The docking results showed that each component had different degrees of binding to each target (Figure 7), and the binding energies with 3CL, ACE2, NA, HA, COX2, and IL-6 were lower than −7.0 kcal/mol.

**Figure 8:**
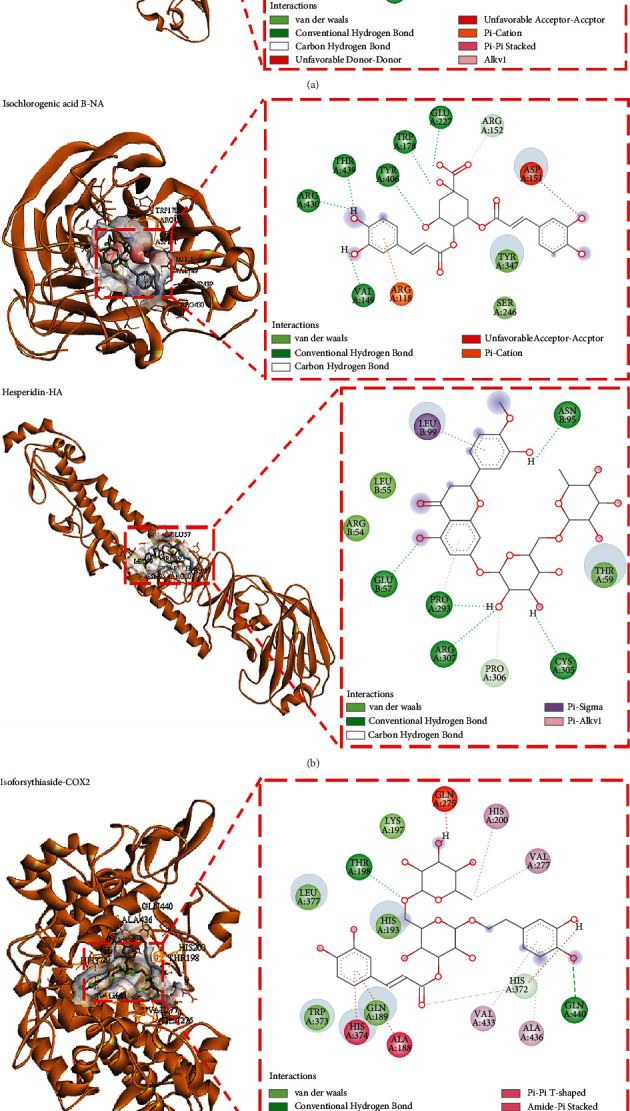
Interactions between the top five components of LHQW and the amino acids of targets related to IVP and NCP with binding energy less than −7.0 kcal/mol. (a) Main components of anti-NCP. (b) Main components of anti-IVP. (c) Main consensus components of anti-IVP and anti-NCP.

The top five components for combination with each target are shown in Figure 8. Amongst them, rutin, isoforsythiaside A, hesperidin, and isochlorogenic acid B were the best components to combine with the six targets. COX2 and IL-6 were the most important common targets of LHQW against viral pneumonia, and the material basis of intervention was the same. However, differences were observed in the key targets of IVP (HA, NA) and NCP (3CL, ACE2) infection, and the material basis of intervention was also emphasised. The main components of anti-IVP are rutin, isoforsythiaside A, and hesperidin, and the main components of anti-NCP were isochlorogenic acid B and forsythin E.

To further analyse the results of each component combined with the targets, this study performed a multi-index comprehensive analysis and multi-index data processing by referring to the general evaluation normalisation method reported in literature [36, 37]. In accordance with Hassan's method (mathematical statistical method that can synthesise various indexes and express the final effect on OD), the docking data of each component and six indicators, namely, 3CL, ACE2, COX2, HA, IL-6, and NA, were processed to calculate the geometric mean of the normalised values of each index, that is, the overall desirability (OD). *D*_imax_ = (*Y*_*i*_ − *Y*_min_)/(*Y*_max_ − *Y*_min_), and OD = (*D*_1_*D*_2_... DK)^1/*k*^ (*k* is the index number) [38, 39]. The results showed that the first five components of the comprehensive index of 3CL, ACE2, COX2, HA, IL-6, and NA from large to small were isoforsythiaside A, hesperidin, isochlorogenic acid B, forsythin E, and quercitrin. The binding energies of isochlorogenic acid B with the six targets, namely, 3CL, ACE2, COX2, HA, IL-6, and NA, were all less than −7 kcal/mol, suggesting that these components play a key role in the treatment of viral pneumonia with LHQW. The results are shown in Table 4.

## 4. Discussion

In this study, 31 main components of LHQW were qualitatively and quantitatively obtained by compiling literature, and 712 component targets were obtained from three databases. A total of 68 common targets for the treatment of viral pneumonia were obtained. The expression of COX2 is abnormal in IVP and NCP [40–43] and mainly distributed in endothelial cells, macrophages, lymphocytes, and so on under inflammatory conditions. The expression of COX2 in lung tissue increases exponentially, and the induced PEG2 accumulates in the lesions, causing the body to produce symptoms, such as redness, swelling, and pain. It also induces a cascade reaction in a vicious cycle, promotes the synthesis of other cytokines, including inflammatory factors (IL-6, TNF-*α*, IL1, etc.), and chemokines (CXCL10, IL-8, etc.), and upregulates the expression of COX2 and NOS2, which induce the synthesis of inflammatory mediators, such as PGE2 and NO [44, 45]. These series of reactions aggravate the inflammatory injury of lung tissue and worsen the patient's condition continuously. In addition, overexpression of COX2 can promote cell proliferation, inhibit apoptosis, inhibit the immune response, and evade immune monitoring [46, 47], such as inhibition of mitochondrial cytochrome C leakage and CASP3/9 activation. This biological process can break the balance between cell proliferation and cell death, creating favourable conditions for pathogen breeding and allowing pathogens to take advantage of it. IL is one of the key common targets, and it includes IL-2, IL-6, and IL-10. IL-6 is produced by monocyte macrophages, Th2 cells, and vascular endothelial cells [48, 49]. NOS2 can promote the synthesis of IL-6 and participate in the inflammatory reaction by producing nitric oxide. MAPK1/3/8/14 and NF-*κ*B, as regulatory factors of the inflammatory response, bind to the IL-6 response element in the promoter of the acute phase protein gene [50, 51]. When the body is activated by cytokines, neurotransmitters, hormones, cell stress, and cell adhesion, they jointly regulate various important physiological and pathological processes, such as inflammatory response, cell growth, differentiation, and stress adaptation. TNF-*α* is an important member of inflammatory cytokines [52, 53] that is mainly composed of activated macrophages, NK cells, and T cells. TNF-*α* can regulate immune cells, and as an endogenous heat source, it can cause fever and interfere with apoptosis. As an early cytokine of viral pneumonia, the expression of TNF-*α* can regulate other proinflammatory factors downstream. An increase in other cytokines stimulates the production of TNF-*α* and triggers the cytokines that are dependent on TNF-*α*. IL-6 and TNF-*α* are the key cytokines [54, 55] for the development of IVP and NCP. TNF-*α*, as an early cytokine, can regulate other proinflammatory factors downstream. An increase in other cytokines stimulates the production of both and initiates the TNF-*α*-dependent cytokine cascade reaction, thus forming a vicious spiral that can cause a cytokine storm and aggravate the patient's condition. The activation and inhibition of these targets not only aggravate the disease but also promote each other, resulting in a vicious cycle that is closely related to the inflammatory injury of the lung; it is also the key target of this study. Therefore, an in-depth study of these targets can help reveal the scientific nature and connotation of the treatment of IVP and NCP.

According to the KEGG pathway enrichment analysis in this study, the IL-17 pathway is the most important signal pathway. IL-17 signal pathways are important to the study of multiple coronaviruses. For example, SARS viruses induce the activation of pattern recognition receptors and IL-17 pathways [56]. The Middle East respiratory syndrome coronavirus (MERS-CoV) is considered a highly pathogenic virus infecting the respiratory tract with high morbidity and mortality. Animal model studies have shown that MERS-CoV infection can cause a strong inflammatory response. After treatment, inflammatory factors, such as TNF-*α*, IL-2, IL-4, IL-15, and IL-17, can be inhibited relatively. IL-17 signalling pathways regulate IL-17 family expression (first discovered by Th17 cells), and by binding to different receptors, they activate downstream signalling pathways JAK-STAT and NFKB to regulate the secretion of other inflammatory factors; IL-17 signalling pathways are also involved in the occurrence and development of lung injury [57, 58]. In recent years, in the outbreaks of the flu viruses and SARS and MERS that killed hundreds of millions of patients, excessive inflammation was observed in patients. Related studies have reported that cytokine storm is the most important and direct cause of lung injury. Another work found that the immune system of patients infected with COVID-19 also exhibits a serious cytokine storm phenomenon [59]. Several studies have confirmed that, in the important pathogenesis of host immune response disorders, Th17/Treg immune imbalance can be a major cause [60, 61], and Th17/Treg immune imbalance is closely related to cytokine storm. Th17 is a proinflammatory cell. The initiation of Th17 differentiation depends on TGF-*β* and IL-6, which can activate signal transduction and the transcription protein pathway by inducing the expression of transcription factors. Th17 mediates inflammatory responses mainly by secreting IL-17, IL-21, IL-2, IL-4, TNF-*α*, and other cytokines [62, 63], but an excessive inflammatory response can cause inflammatory pathological damage to the body. TLR family I transmembrane proteins, which are widely distributed in various cells, can recognise a variety of viruses, mediate the body's antiviral innate immunity and promote the development of acquired immunity; this pathway plays an important role in linking immune and acquired immunity [64]. NF-*κ*B is located at the hub of the downstream signalling pathway in TLR [65]. It can participate in the initiation and regulation of gene expression of many cytokines and inflammatory mediators. After TLR4 binds to its ligand, activated transcription factors enter the nucleus and enhance the transcription of TNF-*α*, IL-1, IL-6, IL-8, and other inflammatory factors; they can also upregulate the expression of COX2 and NOS2, which can induce the synthesis of inflammatory mediators (PGE2 and NO). The accumulation of these cytokines can directly reflect the degree of inflammation. A moderate inflammatory response is a kind of defence reaction of the body, and it is conducive to the clearance of pathogens and the recovery of the body. However, an excessive reaction can cause cascading inflammatory reactions that are difficult to control. TLR and NF-*κ*B may form a complex cycle network with the participation of certain cytokines, which can regulate each other and form a vicious cycle. Therefore, the 20 intervening key targets and their pathways are key to the treatment of viral pneumonia with LHQW.

In addition, the results of molecular docking showed that the 31 main components contained in LHQW key targets had different degrees of combination. In particular, flavonoids and their glycosides and phenylpropanoids had the best binding result. The varied components contained in LHQW could affect multiple pathways by acting on multiple targets to achieve overall intervention in the development of viral pneumonia and overall treatment. Previous studies have found that chlorogenic acid [66], cryptochlorogenic acid [67], forsythin A [68, 69], phillygenin [70], luteolin [71], salidroside [72, 73], and chrysophanol [74] have significant anti-inflammatory effects. The main mechanism is through the significant inhibition of the activation of NF-*κ*B, TLR, MAPK, and other signalling pathways and downregulation of the gene expression of COX2 and iNOS. As a result, the production of proinflammatory factors, such as IL-6, TNF-*α*, and chemokines (MCP-1, CCL2, and CCL10), is reduced to intervene in acute lung injury. Luteolin [75, 76] has a strong inhibitory effect on the proliferation of the influenza virus, but it can also effectively inhibit the expression of inflammatory cytokines and affect the TLR/NF-*κ*B signalling pathway and the expression of apoptosis-related factors (CASP3/8/9, etc.) to reduce the immune damage on the host cells caused by the virus. It also regulates cell apoptosis and plays an antiviral role. Patchouli alcohol [77] has antibacterial and antiviral effects, and it can significantly inhibit the activities of NA and HA, improve the survival rate of mice infected with IAV, and alleviate the symptoms of pneumonia. Its effect is equivalent to oseltamivir. Its mechanism of action is related to the inhibition of the activation of intracellular PI3K/Akt and ERK/MAPK signalling pathways. Chlorogenic acid and its derivatives, such as isochlorogenic acid B and C [78, 79], can effectively inhibit influenza virus infection. Their mechanism of action is to downregulate the expression of viral nucleoprotein and inhibit the activity of NA. They can also effectively reduce the inflammatory reaction and inhibit the production of viral infection. The mechanism is to inhibit the TLR signalling pathway in infected cells and reduce the release of inflammatory factors TNF-*α* and IL-6. Forsythia A [80] can inhibit the replication of the influenza virus, control influenza virus infection, and improve the prognosis. Its mechanism is achieved by inhibiting the activation of TLR, NF-*κ*B, and Th17 signalling pathways. Phillyrin [81] can significantly inhibit the replication of SARS-CoV-2 in vitro and the expression of inflammatory cytokines (TNF-*α*, IL-6, IL-1*β*, CXCL10, etc.) induced by SARS-CoV-2 by regulating the activity of the NF-*κ*B signalling pathway. Therefore, forsythin can be used as a new strategy for coronavirus disease. Research has confirmed that glycyrrhizic acid [82] is similar to a hormone in chemical structure and has a corticoid effect, and it is mainly used to fight a viral inflammatory storm. Forsythin and glycyrrhizic acid are anti-inflammatory drugs. The main difference between them is that glycyrrhizic acid has no immunosuppressive effect. Therefore, glycyrrhizic acid within the safe dose range can resist an inflammatory storm and does not interfere with the ability of the body to resist viruses.

Several studies [83] have found that chrysophanol can inhibit the production of IL-2 by B cells and the expression of the CD40 ligand (CD40L) in activated T cells, thus blocking the NF-*κ*B and MAPK signalling pathways in activated T cells and inhibiting the continuous burst of cytokines. Most of the components reported in these studies are the main components of honeysuckle and forsythia suspense, which belong to the monarch medicine of the LHQW recipe and are the most important components in the treatment of diseases. They clear away heat, detoxify the body, and disperse wind heat. The results of molecular pairing in this study also showed that the main components of honeysuckle and forsythia account for the largest number and are the key to the efficacy of LHQW. The ingredients reported in the above-mentioned studies are basically consistent with the results of the current study, suggesting that the material basis analysis results of this study conform to the law of the monarch, the subject, and TCM to a certain extent. Presently, most studies focus on nonvolatile components, and the few reports on antiviral, anti-inflammatory, and immune regulation of the volatile components of LHQW focus on cooling, expectorant, and antibacterial effects and nasal congestion reduction [84]. The results of molecular docking showed that most of the components of LHQW did not match the new target of IVP and NCP. These results suggest that the contribution of this component to the efficacy of LHQW may not be due to the anti-influenza virus, COVID-19, and antilung inflammation. Volatile components are mainly used to improve nasal congestion, runny nose, cough, expectoration, pharynx, and larynx to create favourable conditions and improve the compliance of patients. All of these compounds are the major components of LHQW and have been extensively studied in the field of influenza infection. Although most of the compounds are not related to NCP, they show good anti-inflammatory, antiviral, and immunoregulation effects, which are closely related to the strategy of treating new crown pneumonia. As the material basis of LHQW, each component exerts a synergistic effect together with the interaction of each target, and these ingredients contribute to the efficacy of LHQW.

An abnormal immune system is the main common point in the occurrence and development of IVP and NCP. The pathway enrichment analysis and biological function annotation in this study showed that the mechanism of LHQW in the treatment of the two diseases is closely related to immune, inflammatory, and viral signalling pathways. Therefore, the mechanism of LHQW treating IVP and NCP could focus on restoring the physiological function of the immune system, reducing the persistent injury of inflammation to the body, inhibiting the invasion and proliferation of the virus, and promoting the apoptosis of the virus.

## 5. Conclusion

LHQW contains 31 main components. Isochlorogenic acid B, isoforsythiaside, forsythoside E, rutin, quercitrin, hesperidin, and other components are the key to the simultaneous treatment of NCP and IVP by LHQW. These components can interfere with signalling pathways, such as IL-17, T cell receptor, Th17 cell differentiation, TNF, toll-like receptor, MAPK, and apoptosis by acting on targets, including 3CL, ACE2, COX2, HA, IL-6, and NA, to realise the anti-inflammatory, antivirus, and immune regulation comprehensive efficacy of LHQW. Although the mechanism of treating IVP and NCP differs and the different contents are mainly reflected in the manner of virus infection and the material basis of antivirus, the main links are closely related to regulating immunity, reducing inflammation, and combating the virus. The process embodies the characteristics of seeking common ground whilst reserving differences in the treatment process of IVP and NCP. It also shows the multicomponent, multitarget, and multichannel advantages of LHQW and provides a research method for the mechanism and substance foundation of the treatment of IVP and NCP.

## Figures and Tables

**Table 1:** Effective chemical constituents of LHQW and ownership of the medicinal materials.

Number	English name	Attribution of medicinal materials
1	Emodin	DH and BLG
2	Rhein	DH and BLG
3	Glycyrrhizic acid	GC and BLG
4	Liquiritin	GC and BLG
5	Chrysophanol	BLG
6	Chlorogenic acid	JYH and LQ
7	4-Caffeoylquinic acid	JYH and YXC
8	Isochlorogenic acid B	JYH
9	Isochlorogenic acid C	JYH
10	Isoforsythiaside	LQ
11	Phillyrin	LQ
12	Forsythoside E	LQ
13	Rutin	GC, BLG, JYH, LQ, MH, and YXC
14	Salidroside	HJT
15	(-)-Phillygenin	LQ
16	Mollugin	JYH
17	Amygdalin	KXR
18	L-Menthol	BHN
19	Patchouli alcohol	GHX
20	Guaiene	GHX
21	*α*-Gurjunene	BHN and MH
22	*α*-Bulnesene	GHX
23	*β*-Patchoulene	GHX
24	*α*-Patchoulene	GHX
25	Quercitrin	YXC
26	Aloe emodin	DH
27	Luteolin	JYH, LQ, and MH
28	Hesperidin	MH
29	Hyperin	GHX, LQ, JYH, and YXC
30	Caffeic acid	JYH and LQ
31	Neochlorogenic acid	JYH and YXC

*Note.* The acronyms in the column “Attribution of medicinal materials” are shown in the first paragraph of “Background.”

**Table 2:** Predicted targets for potential active components of LHQW.

Serial number	Ingredient	Database			Target
		TCMSP	NCBI	SWISS	Total
1	Emodin	35	78	38	151
2	Rhein	30	5	82	117
3	Glycyrrhizic acid	0	69	29	98
4	Liquiritin	22	18	20	60
5	Chrysophanol	30	5	28	63
6	Chlorogenic acid	7	67	39	113
7	4-Caffeoylquinic acid	7	7	39	53
8	Isochlorogenic acid B	2	13	44	59
9	Isochlorogenic acid C	3	15	46	65
10	Isoforsythiaside	0	0	14	14
11	Phillyrin	1	37	7	45
12	Forsythoside E	2	0	21	23
13	Rutin	13	71	21	105
14	Salidroside	0	79	23	102
15	(-)-Phillygenin	27	30	28	85
16	Mollugin	21	41	86	148
17	Amygdalin	19	59	32	110
18	L-Menthol	11	28	32	71
19	Patchouli alcohol	0	0	9	9
20	Guaiene	26	8	3	37
21	*α*-Gurjunene	12	3	5	20
22	*α*-Bulnesene	0	15	0	15
23	*β*-Patchoulene	11	26	10	47
24	*α*-Patchoulene	7	0	11	18
25	Quercitrin	8	69	22	99
26	Aloe emodin	24	78	6	108
27	Luteolin	57	77	46	180
28	Hesperidin	6	78	28	112
29	Hyperin	10	27	30	67
30	Caffeic acid	10	62	64	136
31	Neochlorogenic acid	1	41	39	81

Total number of targets		402	1106	1002	2411
Total number of targets		104	414	341	712

**Table 3:** Topological parameter information table for targets.

Protein name	Gene name	BC	DG
Prostaglandin G/H synthase 2	PTGS2/COX2	0.032	43
Interleukin-6	IL-6	0.067	43
Mitogen-activated protein kinase 14	MAPK14	0.019	38
Tumor necrosis factor	TNF-*α*	0.043	34
Mitogen-activated protein kinase 3	MAPK3	0.046	34
Cellular tumor antigen p53	TP53	0.031	33
Interleukin-2	IL2	0.020	31
Mitogen-activated protein kinase 8	MAPK8	0.022	31
Interleukin-10	IL10	0.020	29
Caspase-3	CASP3	0.032	29
CD40ligand	CD40LG	0.032	29
C-X-C motif chemokine 10	CXCL10	0.022	27
Nuclear factor NF-kappa-B1	NF-*κ*B1	0.023	26
Interleukin-4	IL4	0.009	25
Interleukin-1beta	IL1*β*	0.008	24
Caspase-8	CASP8	0.017	24
Nitric oxide synthase, inducible	NOS2	0.014	23
Interleukin-8	IL8	0.033	23
Mitogen-activated protein kinase 1	MAPK1	0.016	22
Epidermal growth factor receptor	EGFR	0.013	22

**Table 4:** Normalisation and comprehensive evaluation index of molecular docking results.

Serial number	Molecular	3CL	ACE2	COX2	HA	IL-6	NA	Composite index
		Convergence values						
1	Isoforsythiaside	0.95	1.00	1.00	0.69	0.92	0.86	0.90
2	Hesperidin	0.95	0.97	0.77	1.00	0.92	0.84	0.89
3	Isochlorogenic acid B	0.58	0.68	0.81	0.91	1.00	1.00	0.81
4	Forsythoside E	0.86	0.62	0.81	0.78	0.71	0.90	0.78
5	Quercitrin	0.88	0.65	0.74	0.72	0.75	0.88	0.77
6	Amygdalin	0.79	0.73	0.77	0.75	0.63	0.94	0.76
7	Rutin	1.00	0.76	0.88	0.72	0.42	0.78	0.73
8	Luteolin	0.63	0.62	0.63	0.75	0.88	0.90	0.72
9	Isochlorogenic acid C	0.79	0.57	0.5	0.63	0.86	0.65	0.66
10	Hyperin	0.86	0.73	0.60	0.69	0.38	0.80	0.65
11	Chlorogenic acid	0.56	0.57	0.70	0.56	0.67	0.90	0.65
12	DFV	0.67	0.43	0.67	0.72	0.63	0.76	0.64
13	Rhein	0.58	0.57	0.58	0.56	0.67	0.86	0.63
14	Phillyrin	0.72	0.84	0.56	0.66	0.46	0.60	0.63
15	Chrysophanol	0.51	0.49	0.56	0.56	0.46	0.88	0.56
16	Aloe emodin	0.51	0.54	0.49	0.50	0.58	0.80	0.56
17	Emodin	0.53	0.49	0.40	0.63	0.54	0.84	0.56
18	Mollugin	0.56	0.46	0.35	0.59	0.58	0.86	0.55
19	Caffeoylquinic acid 4	0.40	0.30	0.49	0.78	0.67	0.84	0.54
20	Salidroside	0.53	0.30	0.53	0.53	0.67	0.68	0.52
21	Neochlorogenic acid	0.47	0.46	0.37	0.59	0.50	0.86	0.52
22	(-)-Phillygenin	0.51	0.57	0.40	0.72	0.38	0.64	0.52
23	Guaiene	0.14	0.16	0.26	0.47	0.42	0.70	0.30
24	Caffeic acid	0.21	0.08	0.37	0.13	0.33	0.58	0.23
25	*α*-Patchoulene	0.12	0.14	0.12	0.44	0.21	0.68	0.22
26	*β*-Patchoulene	0.12	0.14	0.12	0.44	0.21	0.68	0.22
27	*α*-Bulnesene	0.07	0.08	0.19	0.50	0.13	0.70	0.19
28	*α*-Gurjunene	0.07	0.03	0.07	0.31	0.04	0.66	0.10
29	Patchouli alcohol	0.09	0.11	0.00	0.38	0.08	0.64	0.00
30	Glycyrrhizic acid	0.74	0.43	0.21	0.88	0.17	0.00	0.00
31	L-menthol	0.00	0.00	0.16	0.00	0.00	0.46	0.00

## Data Availability

The original data used to support the findings of this study are included within the supplementary information file.

## Supplementary Materials

Supplementary Materials Supplementary Table 1: molecular docking results. Supplementary Table 2: the top five components with binding energy less than −7.0 kcal/mol. Supplementary Figure 3: interactions between the top five components of LHQW with the amino acids of targets related to IVP and NCP with binding energy less than −7.0 kcal/mol. .
