# Supplementary material for: Exploration of the Mechanism of Lianhua Qingwen in Treating Influenza Virus Pneumonia and New Coronavirus Pneumonia with the Concept of “Different Diseases with the Same Treatment” Based on Network Pharmacology
Source: Evid Based Complement Alternat Med. 2022 Feb 8;2022:5536266. doi: 10.1155/2022/5536266 (PMC8822319; doi:10.1155/2022/5536266)

Supplementary Materials

Supplementary Table 1: Molecular docking results.

Supplementary Table 2: The top five components with binding energy less than −7.0 kcal/mol

Supplementary Figure 3:Interactions between the top five components of LHQW with the amino acids of targets related to IVP and NCP with binding energy less than -7.0 kcal/mol.

Supplementary Table 1: Molecular docking results

Serial number	Molecular	3CL	ACE2	COX2	HA	NA	IL6
		Affinity (kcal/mol)					
1	Isoforsythiaside	-8.8	-9.2	-9.5	-7	-7.2	-6.8
2	Luteolin	-7.4	-7.8	-7.9	-7.2	-7.4	-6.7
3	Guaiene	-5.3	-6.1	-6.3	-6.3	-6.4	-5.6
4	Rutin	-9	-8.3	-9	-7.1	-6.8	-5.6
5	Aloe emodin	-6.9	-7.5	-7.3	-6.4	-6.9	-6
6	Emodin	-7	-7.3	-6.9	-6.8	-7.1	-5.9
7	Patchouli alcohol	-5.1	-5.9	-5.2	-6	-6.1	-4.8
8	Quercitrin	-8.5	-7.9	-8.4	-7.1	-7.3	-6.4
9	α-gurjunene	-5	-5.6	-5.5	-5.8	-6.2	-4.7
10	Amygdalin	-8.1	-8.2	-8.5	-7.2	-7.6	-6.1
11	Chrysophanol	-6.9	-7.3	-7.6	-6.6	-7.3	-5.7
12	Liquiritin	-7.6	-7.1	-8.1	-7.1	-6.7	-6.1
13	Isochlorogenic acid B	-7.2	-8	-8.7	-7.7	-7.9	-7
14	Isochlorogenic acid C	-8.1	-7.6	-7.4	-6.8	-7.2	-5.7
15	Rhein	-7.2	-7.6	-7.7	-6.6	-7.2	-6.2
16	α-patchoulene	-5.2	-6	-5.7	-6.2	-6.3	-5.1
17	Salidroside	-7	-6.6	-7.5	-6.5	-6.3	-6.2
18	4-caffeoylquinic acid	-6.4	-6.6	-7.3	-7.3	-7.1	-6.2
19	Neochlorogenic acid	-6.7	-7.2	-6.8	-6.7	-7.2	-5.8
20	Phillyrin	-7.8	-8.6	-7.6	-6.9	-5.9	-5.7
21	(-)-Phillygenin	-6.9	-7.6	-6.9	-7.1	-6.1	-5.5
22	Forsythoside E	-8.4	-7.8	-8.7	-7.3	-7.4	-6.3
23	chlorogenic acid	-7.1	-7.6	-8.2	-6.6	-7.4	-6.2
24	Hyperin	-8.4	-8.2	-7.8	-7	-6.9	-5.5

25	Glycyrrhizic acid	-7.9	-7.1	-6.1	-7.6	-2.9	-5
26	Mollugin	-7.1	-7.2	-6.7	-6.7	-7.2	-6
27	α-bulnesene	-5	-5.8	-6	-6.4	-6.4	-4.9
28	L-menthol	-4.7	-5.5	-5.9	-4.8	-5.2	-4.6
29	Hesperidin	-8.8	-9.1	-8.5	-8	-7.1	-6.8
30	Caffeic acid	-5.6	-5.8	-6.8	-5.2	-5.8	-5.4
31	β-patchoulene	-5.2	-6	-5.7	-6.2	-6.3	-5.1

Supplementary Table 2: The top five components with binding energy less than −7.0 kcal/mol.

Composition	3CL	ACE2	COX2	HA	NA	IL6
	Rutin	Isoforsythiaside A	Isoforsythiaside A	Hesperidin	Isochlorogenic acid B	
	Isoforsythiaside A	Hesperidin	Rutin	Isochlorogenic acid B	Amygdalin	
−7.0 kcal/mol	Hesperidin	Phillyrin	Isochlorogenic acid B	Glycyrrhizic acid	Forsythoside E	Isochlorogenic acid B
	Quercetin	Rutin	Forsythoside E	Forsythoside E	Luteolin	
	Forsythoside E	Hypericin	Hesperidin	4-caffeoylquinic acid	Chlorogenic acid	

Supplementary Figure 3:Interactions between the top five components of LHQW with the amino acids of targets related to IVP and NCP with binding energy less than -7.0 kcal/mol.

Isoforsythiaside-3CL

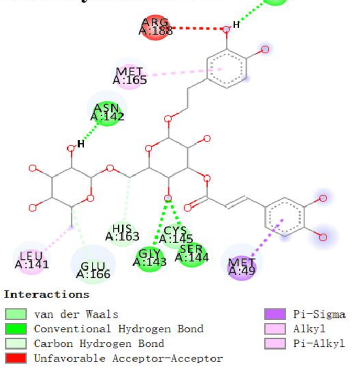

Hesperidin-3CL

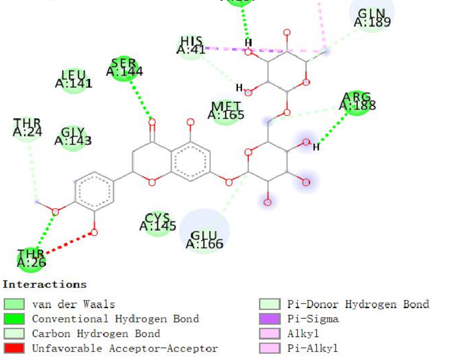

Quercetin-3CL

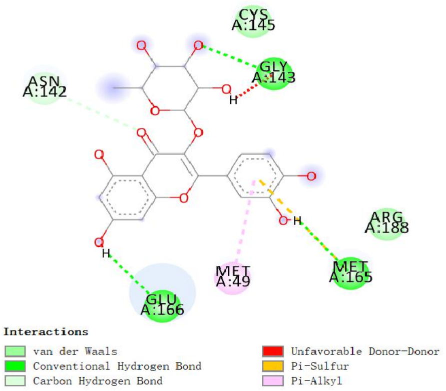

Forsythoside E-3CL

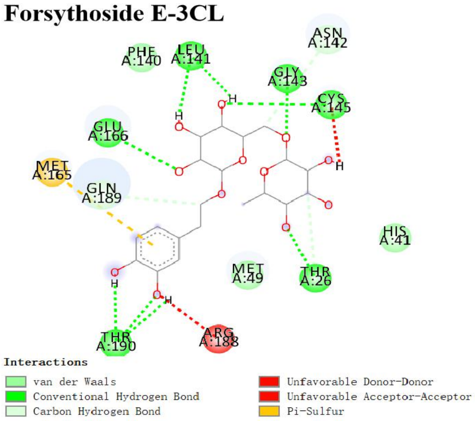

Hesperidin-ACE2

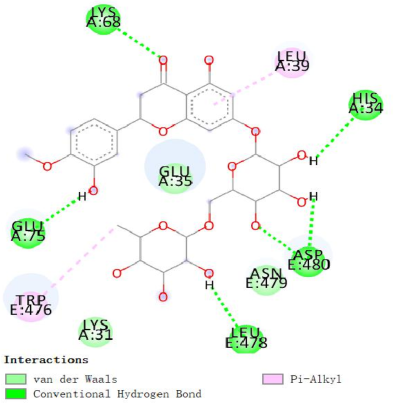

Phillyrin-ACE2

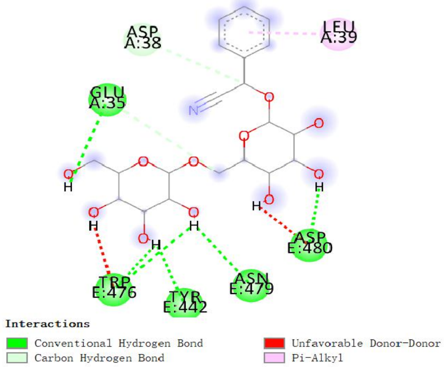

Rutin-ACE2

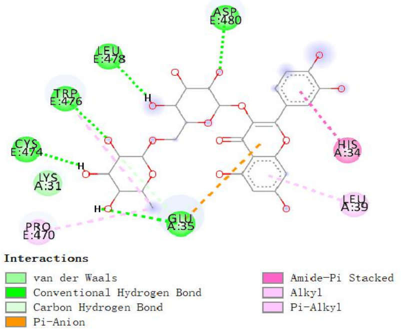

Hypericin-ACE2

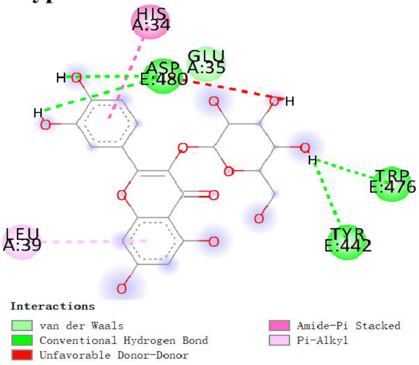

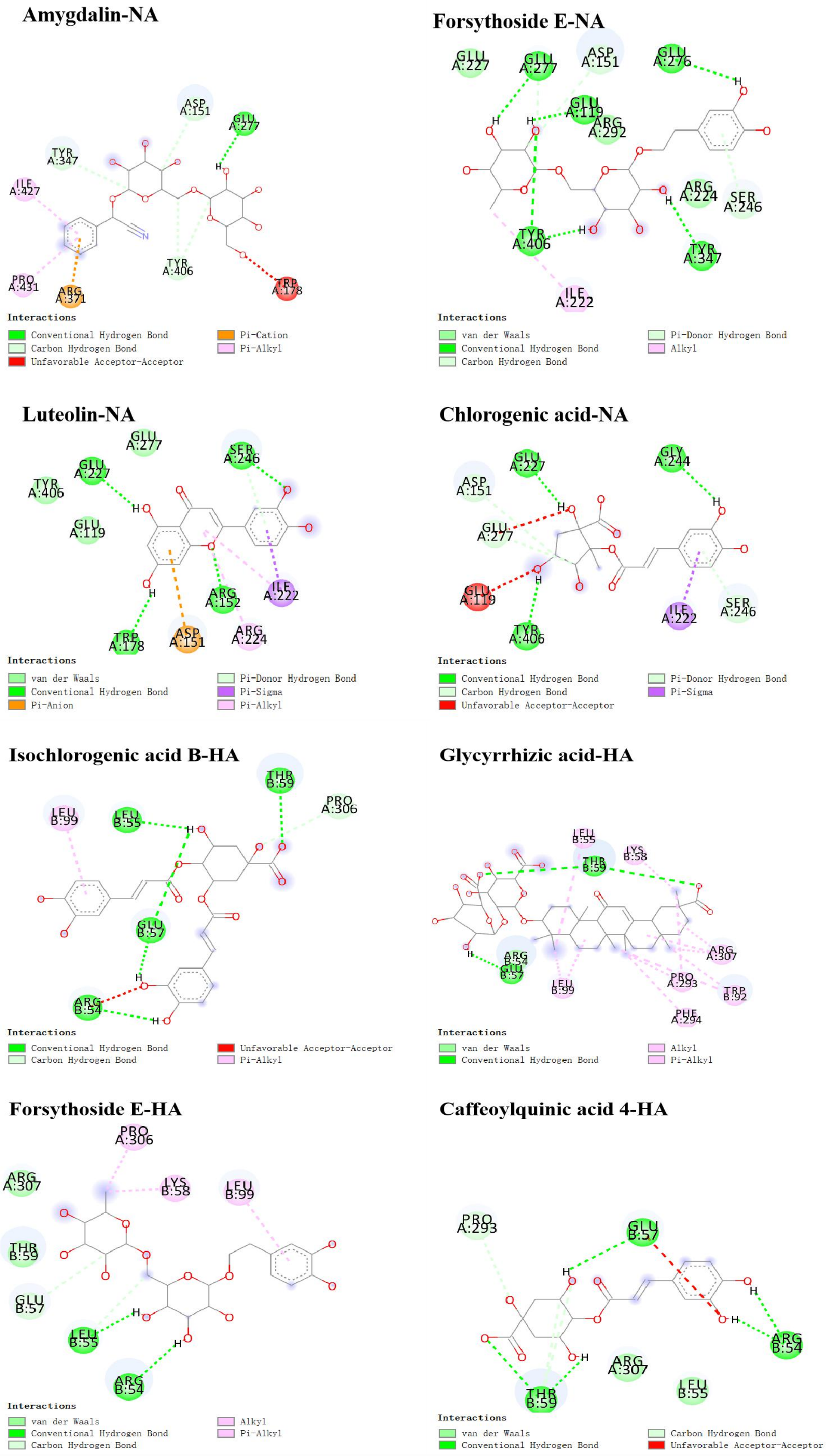
Amygdalin-NA
Interactions
Conventional Hydrogen Bond
Carbon Hydrogen Bond
Unfavorable Acceptor-Acceptor
Pi-Cation
Pi-Alkyl
Forsythoside E-NA
Interactions
van der Waals
Conventional Hydrogen Bond
Carbon Hydrogen Bond
Pi-Donor Hydrogen Bond
Alkyl
Luteolin-NA
Interactions
van der Waals
Conventional Hydrogen Bond
Pi-Anion
Pi-Donor Hydrogen Bond
Pi-Sigma
Pi-Alkyl
Chlorogenic acid-NA
Interactions
Conventional Hydrogen Bond
Carbon Hydrogen Bond
Unfavorable Acceptor-Acceptor
Pi-Donor Hydrogen Bond
Pi-Sigma
Isochlorogenic acid B-HA
Interactions
Conventional Hydrogen Bond
Carbon Hydrogen Bond
Unfavorable Acceptor-Acceptor
Pi-Alkyl
Glycyrrhizic acid-HA
Interactions
van der Waals
Conventional Hydrogen Bond
Alkyl
Pi-Alkyl
Forsythoside E-HA
Interactions
van der Waals
Conventional Hydrogen Bond
Carbon Hydrogen Bond
Alkyl
Pi-Alkyl
Caffeoylquinic acid 4-HA
Interactions
van der Waals
Conventional Hydrogen Bond
Carbon Hydrogen Bond
Unfavorable Acceptor-Acceptor

Amygdalin-NA

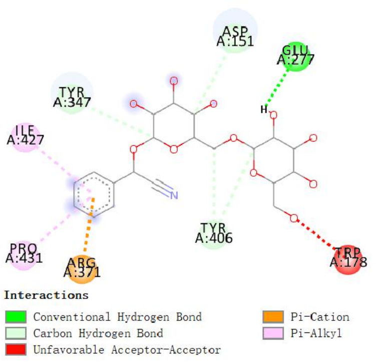

Forsythoside E-NA

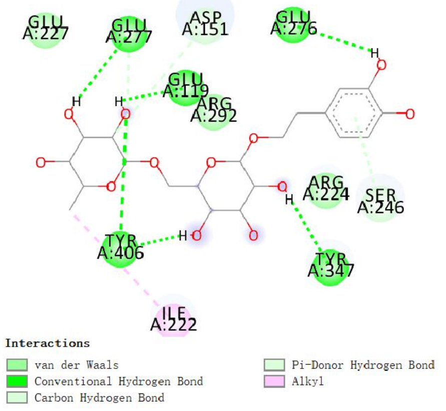

Luteolin-NA

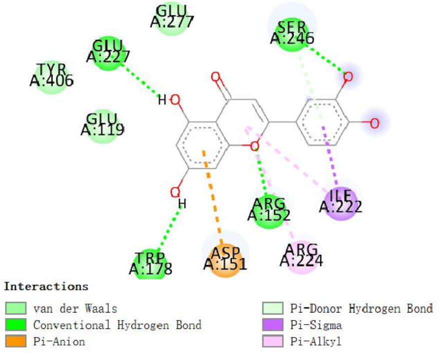

Chlorogenic acid-NA

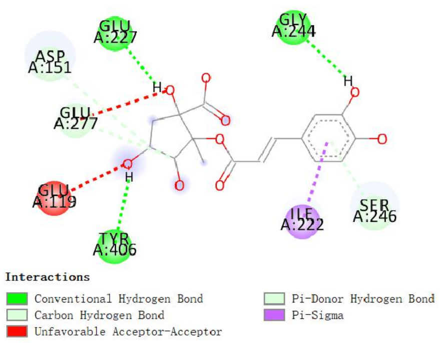

Rutin-COX2

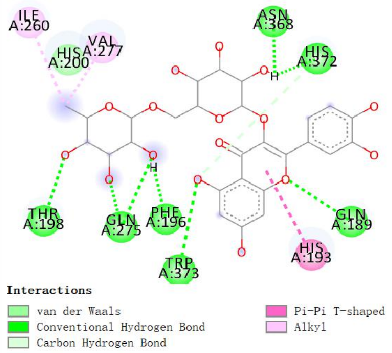

Isochlorogenic acid B-COX2

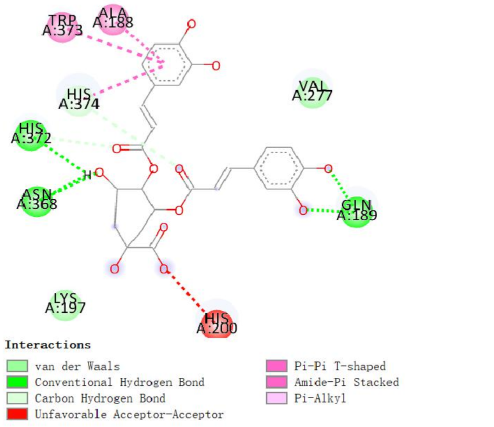

Forsythoside E-COX2

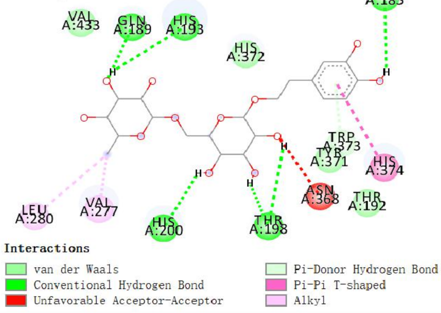

Hesperidin-COX2

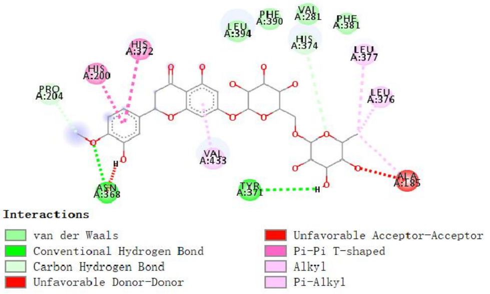

Supplement: Supplementary Materials — Supplementary Table 1: molecular docking results. Supplementary Table 2: the top five components with binding energy less than −7.0 kcal/mol. Supplementary Figure 3: interactions between the top five components of LHQW with the amino acids of targets related to IVP and NCP with binding energy less than −7.0 kcal/mol. . [file 5536266.f1.pdf]
